# Hearing the Sound in the Brain: Influences of Different EEG References

**DOI:** 10.3389/fnins.2018.00148

**Published:** 2018-03-13

**Authors:** Dan Wu

**Affiliations:** ^1^School of Computer and Information Technology, Beijing Jiaotong University, Beijing, China; ^2^The Key Laboratory for NeuroInformation of Ministry of Education, University of Electronic Science and Technology of China, Chengdu, China

**Keywords:** brainwave music, EEG, reference electrode, reference electrode standardization technique (REST), scale-free

## Abstract

If the scalp potential signals, the electroencephalogram (EEG), are due to neural “singers” in the brain, how could we listen to them with less distortion? One crucial point is that the data recording on the scalp should be faithful and accurate, thus the choice of reference electrode is a vital factor determining the faithfulness of the data. In this study, music on the scalp derived from data in the brain using three different reference electrodes were compared, including approximate zero reference—reference electrode standardization technique (REST), average reference (AR), and linked mastoids reference (LM). The classic music pieces in waveform format were used as simulated sources inside a head model, and they were forward calculated to scalp as standard potential recordings, i.e., waveform format music from the brain with true zero reference. Then these scalp music was re-referenced into REST, AR, and LM based data, and compared with the original forward data (true zero reference). For real data, the EEG recorded in an orthodontic pain control experiment were utilized for music generation with the three references, and the scale free index (SFI) of these music pieces were compared. The results showed that in the simulation for only one source, different references do not change the music/waveform; for two sources or more, REST provide the most faithful music/waveform to the original ones inside the brain, and the distortions caused by AR and LM were spatial locations of both source and scalp electrode dependent. The brainwave music from the real EEG data showed that REST and AR make the differences of SFI between two states more recognized and found the frontal is the main region that producing the music. In conclusion, REST can reconstruct the true signals approximately, and it can be used to help to listen to the true voice of the neural singers in the brain.

## Introduction

The electroencephalogram (EEG), one of the most useful technologies for brain research, has been recommended for its non- invasiveness and high time resolution. However, the obtained EEG information is almost always presented as complicated visual images or waveforms. If brain waves could be heard after translation by a proper sonification rule, we may be able to directly “perceive” brain activity and its variations using our auditory system. Some studies investigate that the rhythm of human breathing, movements, and even synchronization follow the scale free law (Torre and Wagenmakers, 2009; Hennig, 2014), which is shared with music (Manaris et al., 2005; Levitin et al., 2012; Liu et al., 2013). In that way, the brain activities recorded from the scalp may be due to neural singers in the brain.

To hear sound of the brain, many strategies are adopted by researchers in different fields, from neuroscience to music composition. Because the main frequency of EEG is so low that it cannot be hear directly, the most basic method is parameter mapping, which translates a few parameters of EEG to the characteristic parameters of music. In some early works, for example, the earliest attempt to hear brainwaves as music (Adrian and Matthews, 1934) and a concert called “Music for Solo Performer” (Rosenboom, 1976), the amplitude of the alpha waves from EEG signals were utilized as the driving sources of the musical sound. In the 1990s, various new music generating rules were created from digital filtering or coherent analysis of EEG (Rosenboom, 1997). To date, parameter mapping is the most popular method and widely used (Rosenboom, 1976; Hinterberger and Baier, 2005), because it not only provides a sensitive way to detect the small variations in the amplitude and frequency of brain waves that are ignored by conventional EEG technique in real time (Väljamäe et al., 2013), but also can indicate some essential features, i.e., the scale free law, followed by both EEG and music (Wu et al., 2009). Another typical method was the event triggering, which utilizes specific events such as interictal epileptic discharges as triggers for the beginning of supposed music tones or other sound events (Baier et al., 2007).

In fact, more than one strategy is used for the music generation in real systems. The musical application of Brain Computer Interface (BCI) can represent the connections between mental states and music (Miranda and Brouse, 2005; Miranda, 2010; Wu et al., 2010), and detect users' current affective states significantly (Daly et al., 2016). To express the activities of different brain regions, several instruments were used to represent different brain regions and that just make the brain like an orchestra (Hinterberger and Baier, 2005); the voice or music for the left and right channels were deduced by the activities of the respective spheres (Wu et al., 2014). Deriving a quartet from multi-channel EEGs with artistic beat and tonality filtering, we can harmonically distinguish the different states of the brain activities (Wu et al., 2013). The combination of EEG and fMRI provided more information of the brain which can be heard (Lu et al., 2012). Listen to the music or sound of the brain is a good way for investigating the brain activities, but a crucial factor is that the reality and accuracy of the sound we heard.

During EEG scalp recording, one of the most fundamental points which influences the accuracy of data, is the reference choosing, and that is a very attractive question in brain electrophysiology research. Using an appropriate reference is essential for data collecting and analyzing, because the potential difference only can be measured between two points, the objective electrodes and the reference (Geselowitz, 1998). Several different types of reference, including the vertex reference (CZ), the linked mastoids reference (LM), the average reference (AR), and the left mastoid reference (L), are currently used for EEG measurement. However, all of these references may lead to an undesired temporal bias since no neutral point exists on the body surface. Thus, the reference signal itself may involve physiological dynamic processes that will inevitably influence the data. Previous studies have examined the effects of reference choice on EEG data using several methods, including the estimation of the effect of head surface on recordings using AR (Junghöfer et al., 1999; Yao, 2017), the examination of coherence and reference signals (Nunez et al., 1997; Essl and Rappelsberger, 1998). To entirely resolve the problems involved in using body surface points for referencing, a reference with neutral potential is required. Theoretically, a point at infinity is far from brain sources, and has an ideally neutral potential. In 2001, Yao proposed a “reference electrode standardization technique (REST)” to approximately transform EEG data recorded with a scalp point reference to recordings using an infinity reference (the software for REST transformation can be downloaded at http://www.neuro.uestc.edu.cn/rest). In recent years, the REST has been quantitatively validated through simulation studies with assumed neural sources in both a concentric three-sphere head model (Yao, 2001) and a realistic head model (Zhai and Yao, 2004). These studies have shown that data referenced with REST are more consistent with physiology than data referenced using traditional scalp references. This has been shown using a variety of techniques, including EEG spectral imaging (Yao et al., 2005), EEG coherence (Marzetti et al., 2007), brain evoked potentials (EP) and spatiotemporal analysis (Yao et al., 2007), default network (Qin et al., 2010).

However, if we believe that brain activities are musical, the influence of reference to scalp EEG recordings definitely will affect the music of EEG. Especially, our scale free music is supposed to be an objective reflection of the physiological signal, we need to have the true objective EEG signal without non-zero reference effect etc. In this work, we assume that the neural activities are musical, for the EEG shows 1/f fluctuation (Leistedt et al., 2007; Tomasi et al., 2017) the same as the pitch, rhythm and consonance of the music (Manaris et al., 2005; Levitin et al., 2012; Wu et al., 2015). Then we primarily use classical music as the source of EEG, and forward calculate the scalp “potential” of them, and to see what's the effect of the reference on the scalp potential, the music of the brain. In this study, the tested reference are REST, average reference (AR), and linked mastoids (LM), and the evaluation metrics is the relative error between the true scalp potential/music and the re-reference potential/music. Finally, a real data music was comparatively investigated.

## Materials and methods

### Music materials for simulation

Since the neural activities in the brain may be musical, we use some classical music pieces as the EEG source to test the scalp potential. The music piece *Two Part Inventions* (*BWV 772)*, which wrote by composer Johann Sebastian Bach (1685–1750), was used as one-source and two-source signals for simulation. The music was firstly translated from MIDI format to waveform before using as the one source signal in the brain.

The next, the original MIDI file was separated into two parts according to the polyphonic principle, translating into two series of audio waves, and then used as two source signals for simulation. BWV772 was a typical polyphonic music style, which contained two related independent melody and the two parts or two voices were skillfully interweaving in the work according to the polyphonic principle.

**Figure 1:**
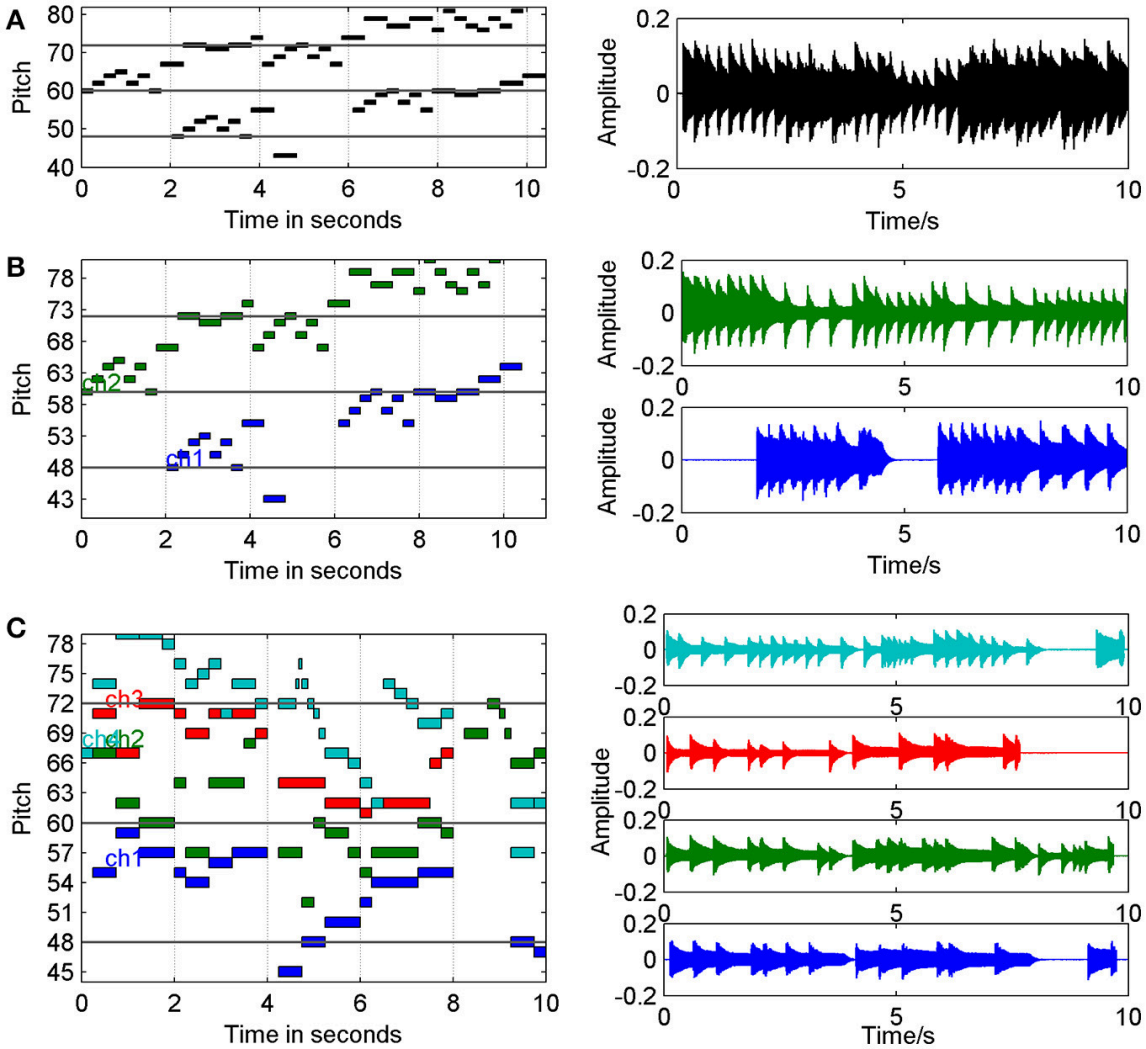
Music materials for simulation in MIDI and waveform format (10 s for example). **(A)** MIDI and waveform of Bach, BWV 772, with one voice. **(B)** MIDI and waveform of Bach, BWV 772, with two voices. **(C)** MIDI and waveform of Mozart, K387, with four instruments.

Another classical music piece used in the simulation was one of the famous works of Wolfgang Amadeus Mozart (1756–1791), *Quartet No.14 in G for strings (K387)*, which was used as four-source signals. A quartet was wrote for four instruments, thus we can easily divide the work into four simultaneous parts in the MIDI sequences, and then translate them into four channel waveforms, finally put these signals in the head modal as four sources. Figure 1 has shown 10 s of the MIDI and waveform format files of these music pieces.

### Simulation process

Before the simulation, all the music pieces were prepared in waveform format (the file name was ^*^.wav) with sampling rate 44,100 Hz, and it can be read into MATLAB as matrixes. The music data were represented as *M*, which was a 1^*^N, 2^*^N or 4^*^N matrix for the three cases in Figure 1. Here *N* was the time point length of the music and 1, 2, or 4 represented the number of the music parts, also of the sources. For simulation, the forward EEG calculation is given by

(1) $$\begin{array}{r} V=GS \end{array}$$

where *G* is the transfer matrix referenced at infinity, only dependent on the head model, source configuration and electrode montage; *S* is the distributed source; and *V* is the scalp EEG recording with a reference at infinity generated by *S*. Scalp noise is not considered in this model and is assumed to be zero. To simulate a source in a brain model, the transfer matrix (*G*) must be established by using the location information of an electrode cap system. In this work, with known electrode location, source locations, and a three-layer spherical head model, the transfer matrix *G* can be obtained. With known source temporal processes, matrix *S*, it can be further assumed that the sources are all radial dipoles (Yao, 2000). Therefore, the signals on the scalp (*V*) were obtained by forward modeling Equation (1), and *V* was the standard signals with reference at infinity in the simulation.

In this study, the head model for all cases was a three-concentric-sphere model. The normalized radii of the three concentric spheres were 0.87 (inner radius of the skull), 0.92 (outer radius of the skull) and 1.0 (radius of the scalp). The normalized conductivities were 1.0, 0.0125, and 1.0 for the brain, skull and scalp, respectively. The center of the spheres was defined as the coordinate origin. The x-axis was oriented from the origin to the direction of the right ear, and the y-axis was oriented in the posterior–anterior direction. The z-axis was oriented from the origin to the vertex.

With known simulated *V* with zero reference, or actual scalp recordings *V* with one point such as Cz or left ear, etc. as reference, it is easy to translate them to data with any one of the three references: REST, average reference (AR), and linked mastoids reference (LM) (Yao, 2001). Here, we adopt the free software REST to do it (www.neuro.uestc.edu.cn/rest). And we chose a classical 10-20 system with 32 electrodes.

### The comparison of three references

To compare the influences of the REST, AR, and LM, we should first calculate the standard scalp signals at different electrodes according to the sources, and then translate into the three references occasions. For the simulation, signals on the scalp were the music with distortion by the references, so that we can listen to these music pieces for further investigation. In order to quantify the differences, relative error and correlation coefficients between the standard signal and the three re-referenced signals were calculated. Relative error demonstrated the relative value difference between the estimated signals and the standard at every time point, while the correlation coefficients can explain the variations in a holistic view. For all the simulations in this study, the head model was a three-concentric-sphere model mentioned in section Simulation Process. The locations of sources were used as another factor in the comparison. For one source situation, we tested 300 locations, which picked up from the 3,000 locations according to the positions of the head modal proposed in transfer matrix calculating (Yao, 2000). And for two sources situation, we tested 380 pairs of locations that means each source was put in 20 locations, respectively. At last, 360 pairs of location for four sources were performed.

### Real EEG to brainwave music with different references

We also used real EEG data with different references to generate brainwave music. The data was recorded in an orthodontic pain control experiment (Huang et al., 2016). This study was approved by the Ethics Committee of the West China Hospital of Stomatology. All subjects gave written informed consent in accordance with the Declaration of Helsinki. In the experiment, 24 subjects (23 ± 5 years old) were right-handed, had mild dental crowding. There were two groups (each group 12 subjects): subjects group 1 (brain music group) were just listening the brainwave music of their own; those in group 2 (control group) without any interfering. The brainwave music used in group 1 was generated from the EEG before the subjects been treatment, in that time they did not suffer the pain. The EEG data adopted in this study were recorded on the second day after the initial archwire placement for the subjects.

EEG signals were recorded by the SymTop EEG system (SymTop Instrument, Beijing, China) with 16 Ag/AgCl surface electrodes fixed in a cap at the standard positions according to the 10–20 system. The EEG was referenced to the mean of the signals recorded at the participants' mastoids (LM). Impedances were kept below 5 kΩ. EEG signals were sampled at 1000 Hz, 0.3–45 Hz band-pass filter. These parameters were used for all the EEG recordings in this study. The data, recording in the second day after the pain beginning, were chosen for translating into music pieces to compare the differences of the three references.

The method for brainwave music generation in this paper was proposed in 2013 (Wu et al., 2013), which deriving a quartet from multi-channel EEGs with artistic beat and tonality filtering. EEG data from multiple electrodes were first translated into MIDI sequences by scale free brainwave music method (SFBM) (Wu et al., 2009), respectively. Then, these sequences were processed by a beat filter which adjusted the duration of notes in terms of the characteristic frequency. And the sequences were further filtered from atonal to tonal according to a key defined by the analysis of the original music pieces. The note which lasted for the longest time in the music was determined as the main note of a certain key, and after that the musical filter would chose the notes which supported the key from the original sequences produced by the EEG.

The original reference of the recorded EEG data was the LM reference, and we changed it to the AR and REST reference for comparison. The EEG of three different references were translated into music pieces, respectively. The features of music, such as pitch, tempo, note duration, scale free exponent of pitch, and the weight of every electrode in the music generation were calculated.

## Results

### Single source simulation

The signal used as a single source in the simulation was *BWV 772* (Figure 1A) that composed by Bach. The source was put in 300 different locations of the head modal, and I calculated the relative errors between the standard signals and the re-reference. The average relative errors of the 300 locations were 0.72 ± 2.88 (REST), 4.93 ± 16.69 (AR), and 4.39 ± 7.79 (LM). The one way ANOVA were used to test the differences between the three groups (*p* < 0.01). And the results of *post-hoc* test showed significant differences between REST and AR, REST, and LM (Tukey's honestly significant difference criterion).

**Figure 2:**
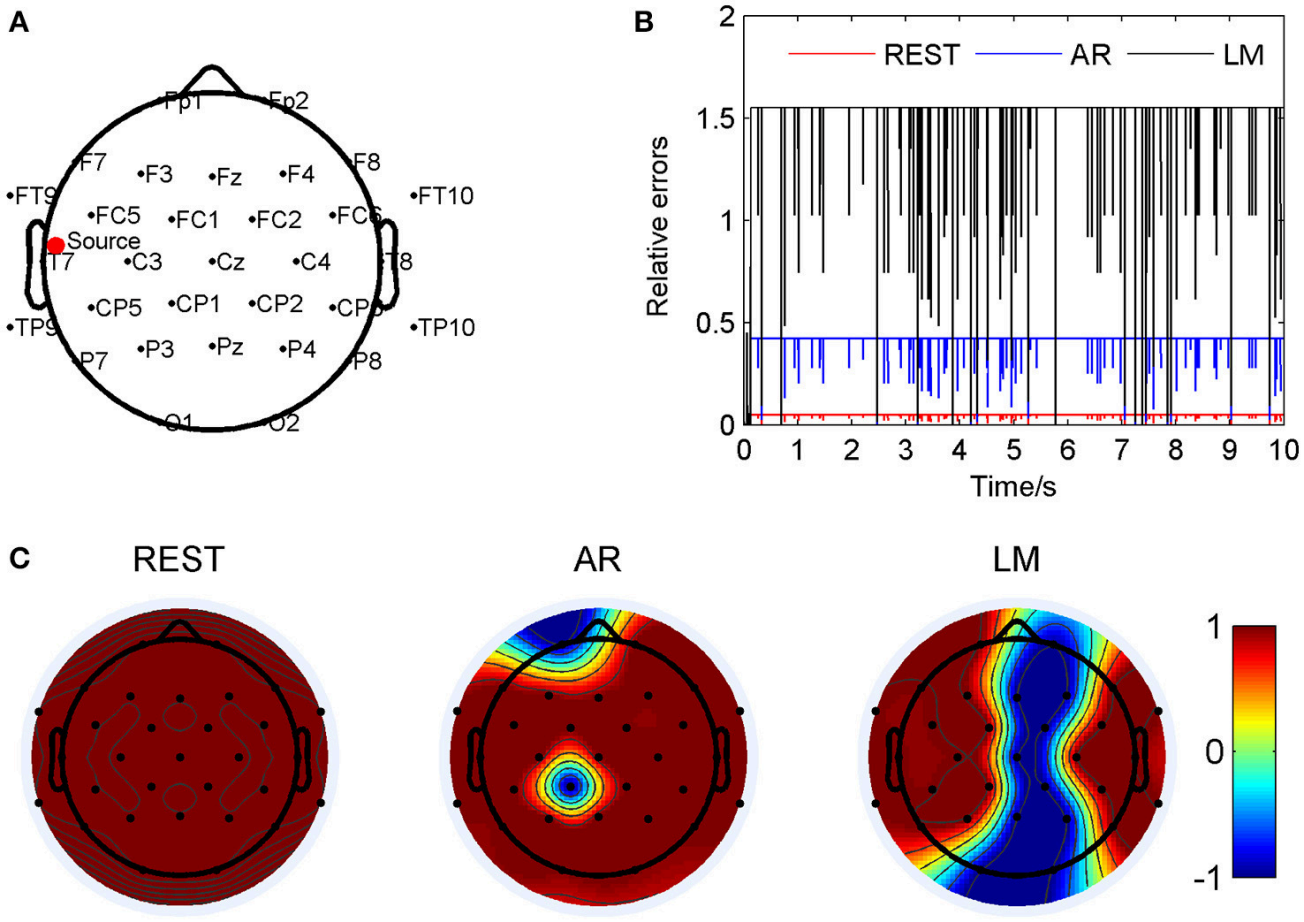
Comparison of the three reference electrode methods for the one source of BWV772 shown in Figure 1A. **(A)** The location of the source is (−0.65674, −0.06352, 0.56552). The red dot indicates the projected position of the source on the scalp surface. **(B)** Relative errors of the music signals with three references compared to the standard signals. **(C)** Correlation coefficients of three references with the standard signals.

As an example, the coordinate of one source location was (0.087, 0.859, 0.097), and marked by a red dot in Figure 2A. Relative errors of the three different electrode references were calculated compared to the standard signals and showed in Figure 2B. In this location, the REST method showed a significant smallest relative errors, about 0.049 ± 0.006; next was the AR, 0.41 ± 0.05; and the LM was the highest, 1.52 ± 0.20, averagely. Figure 2C showed correlation coefficients between the standard signals and the three references, respectively. It was obvious that all the three re-referenced methods highly correlated to the standard signal, especially the REST, the coefficients of all the 32 electrodes were 1. For AR and LM, coefficient values for several electrodes were −1, which means just a phase shift during the process. For AR, its overbearing assumption, that the average of the whole recordings would be zero, definitely result part of the scalp positive and other part negative, for LM, the electrodes with signals smaller than the average of the two ears will be negative, and the larger one will be positive if the actual value of LM is positive relative to infinity. Four music pieces, including the standard signal, the signal from electrode CP1 on the scalp of REST, AR, and LM, were provided in the Supplementary Material. It was easy to find that the standard signal and REST music sounded the same, while the AR and LM music had small volume compared to the formers.

### Two sources simulation

Testing 20^*^19 pairs of sources, which were chosen from all the 3,000 locations according to the head modal, I found that the waveform/music showed distinct differences in the three references. The average relative errors of the 380 pairs were 0.17 ± 0.26 (REST), 2.29 ± 1.71 (AR), and 4.12 ± 3.18 (LM). The one way ANOVA were used to test the differences between the three groups (*p* < 0.01). And the results of *post-hoc* test showed significant differences between REST and AR, REST, and LM, AR and LM (Tukey's honestly significant difference criterion). Here two location pairs were shown because of their typical distributions for the AR and LM references, respectively.

**Figure 3:**
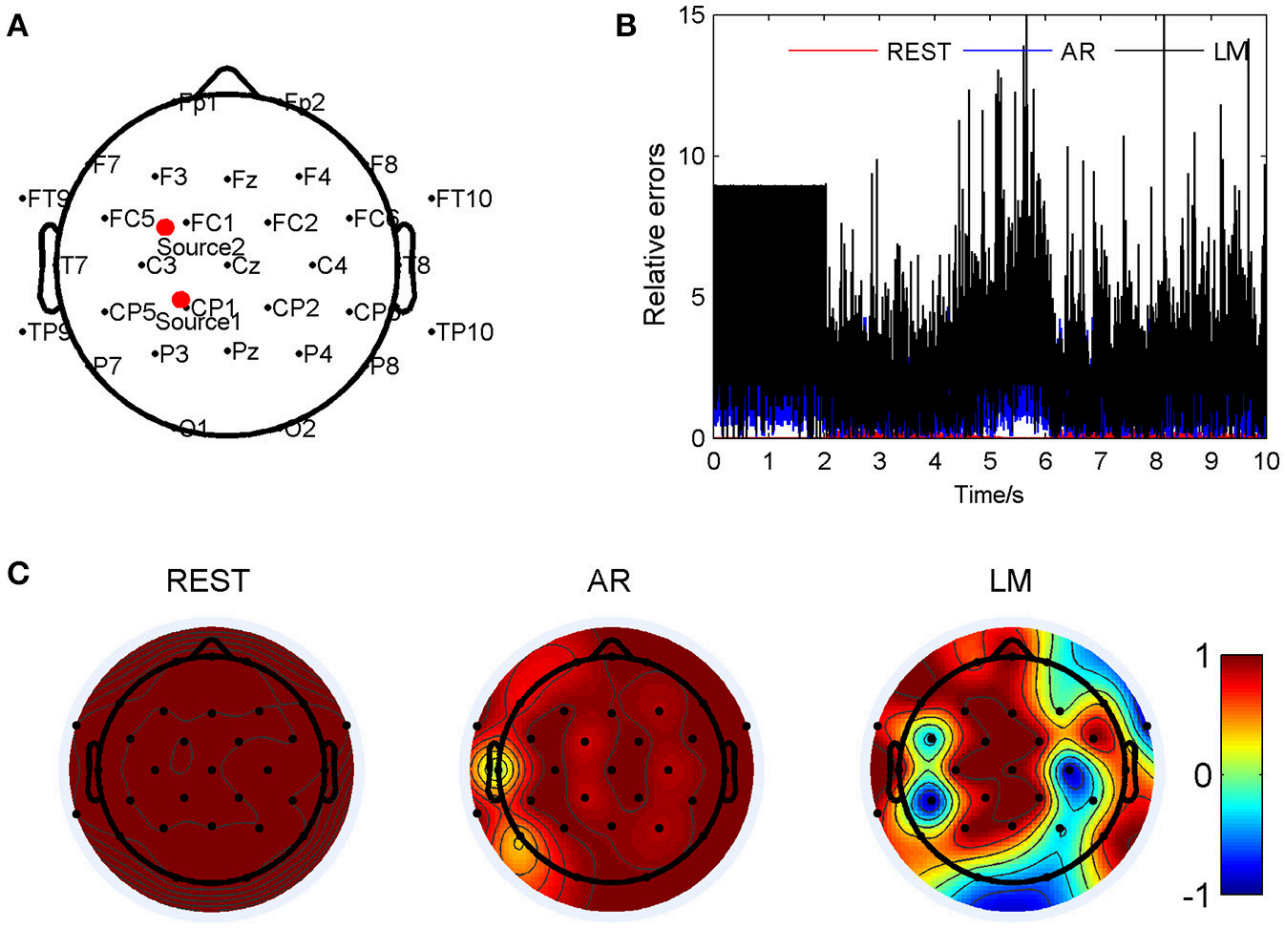
Comparison of three reference electrode methods for the two sources of BWV 772 shown in Figure 1B. **(A)** The location of the sources are (−0.264, 0.352, 0.750) and (0.278, 0.457, 0.689). The red dots indicate the projected positions of the source on the scalp surface. **(B)** Relative errors of the music signals with three references compared to the standard signals. **(C)** Correlation coefficients of three references with the standard signals.

Figure 3 has shown the situation with source locations at (-0.264, 0.352, 0.750) and (0.278, 0.457, 0.689), so that AR reference was deflected from the standard signal mostly. The two sources were marked as Source 1 and Source 2 in Figure 3A. The relative errors of the three different electrodes were calculated compared to the standard signals and showed in Figure 3B. For the location pair, the REST method showed a significant smallest relative errors, about 0.053 ± 0.060; next was the AR, 1.38 ± 0.96; and the LM was the highest, 2.74 ± 2.14, averagely. Figure 3C showed correlation coefficients between the standard signals and the three references, respectively. In this situation of source pair, it was obvious that REST was almost the same with the standard signals; the coefficient of every electrode was 1. For AR reference, most regions were accordant with the standard, but at electrode T7 was 0.05, and P7 was 0.35. For LM reference, the electrodes, T7 was 0.99, but P7 was 0.33, CP5 was −0.97, also showed a low correlation to the standard signal. Four music pieces, lasting 30 s, from the electrode T7 on the scalp, including the standard signal, REST, AR, and LM, were provided in the Supplementary Material. To listen to these music, it can be found that in the standard signal and REST music, the volume of channel two was larger than channel one, while in AR music, channel one was larger. LM music sounded a little larger volume than the standard.

**Figure 4:**
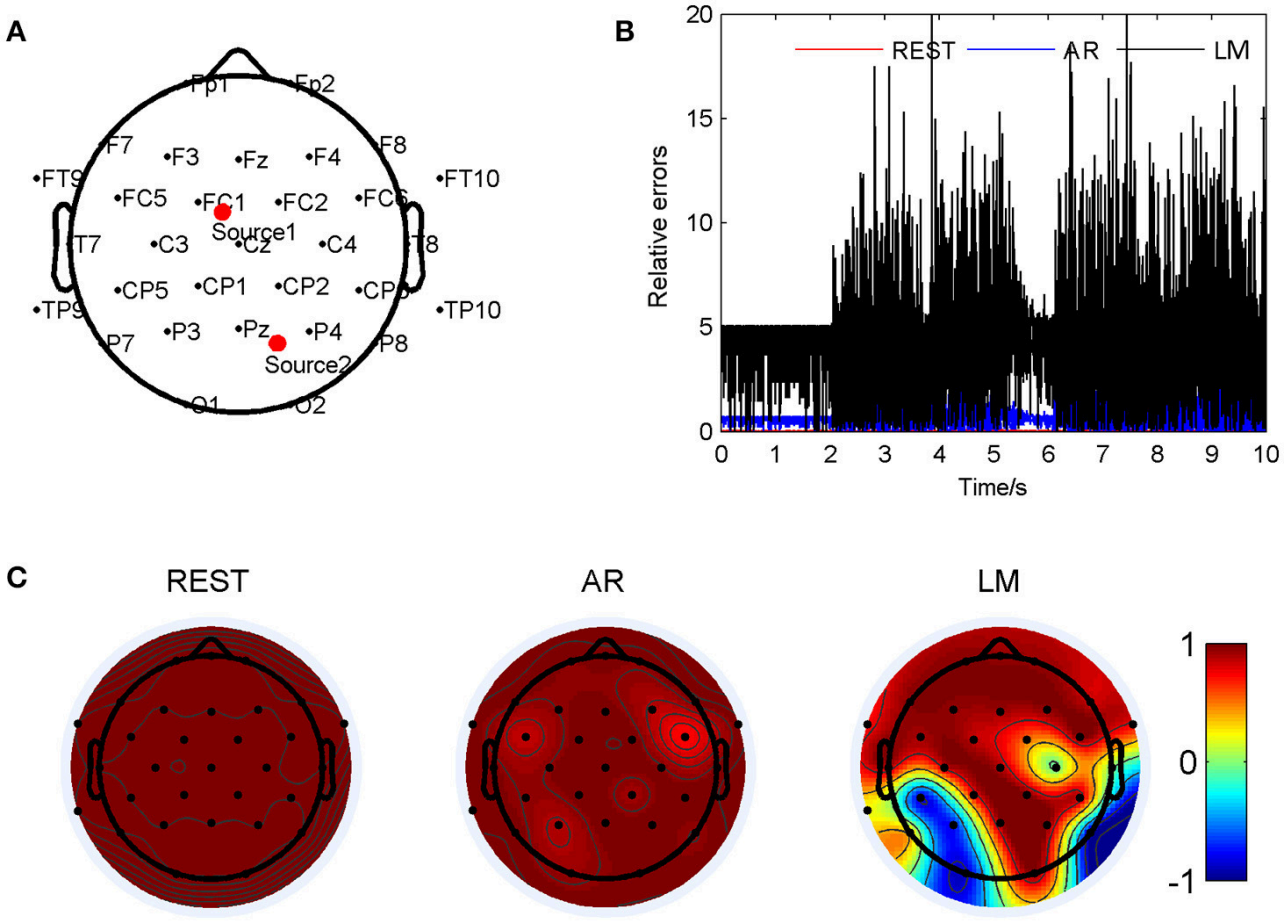
Comparison of the three reference electrode methods for the two sources of BWV 772 shown in Figure 1B. **(A)** The location of the sources are (0.254, 0.124, 0.822) and (−0.678, −0.267, 0.473). The red dots indicate the projected positions of the source on the scalp surface. **(B)** Relative errors of the music signals with three references compared to the standard signals. **(C)** Correlation coefficients of three references with the standard signals.

Figure 4 has shown the situation when the two source were at (0.254, 0.124, 0.822) and (−0.678, −0.267, 0.473) so that LM reference was deflected from the standard signal mostly. The two sources were marked as Source 1 and Source 2 in Figure 4A. For such location pair, the REST method showed a significant smallest relative errors, about 0.036 ± 0.017; next was the AR, 0.90 ± 0.67; and the LM was the highest, 4.13 ± 2.24, averagely. These results have shown in Figure 4B. The correlation coefficients between the standard signals and the three references were shown in Figure 4C, which demonstrated that the REST reference was the same as standard signal. The LM reference dedicated low correlation coefficients in many electrodes, especially in the occipital of the cap. For example, P8 was −0.89 and O1 was −0.87. Compared to LM, the AR reference was the almost high correlated with the standard, P8 of AR was 0.95, O1 was 0.93, but in FC5 and FC6, the correlation was down to 0.85 and 0.71. By listening the music from the electrode P8 on the scalp, it was observed that channel one in LM music was hardly been heard compared to the other three pieces.

### Four sources simulation

Three hundred and sixty pairs of electrodes which were chosen from all the 3,000 locations of the head modal were tested in the four source simulation, and the average relative errors were 0.07 ± 0.03 (REST), 0.77 ± 0.13 (AR), and 2.28 ± 0.75 (LM), respectively. The one way ANOVA were used to test the differences between the three groups (*p* < 0.01). And the results of *post-hoc* test showed significant differences between REST and AR, REST, and LM, AR, and LM (Tukey's honestly significant difference criterion).

**Figure 5:**
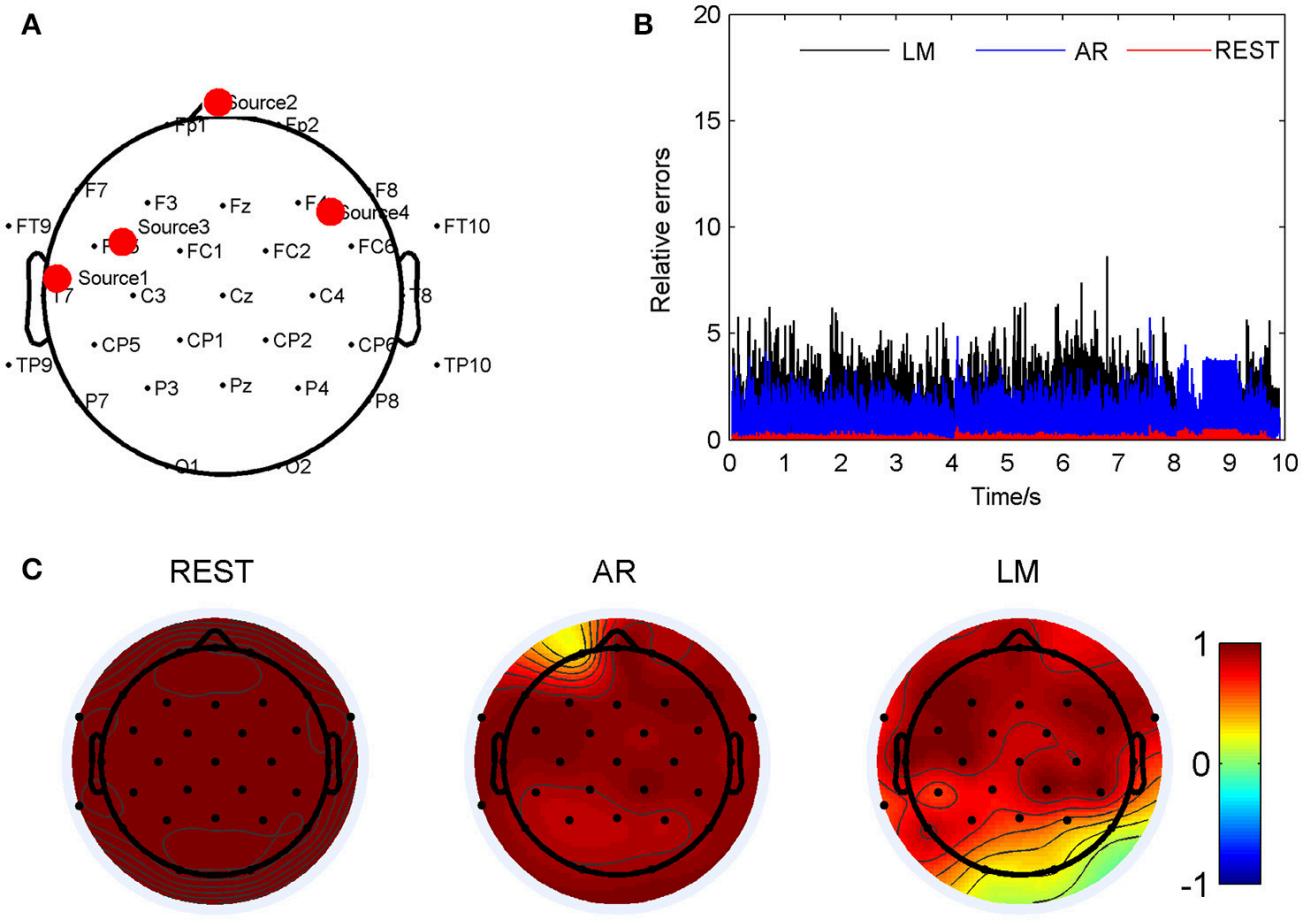
Comparison of the three reference electrode methods for the four sources of K387 shown in Figure 1C. **(A)** The location of the sources are (0.087, 0.859, 0.097), (0, 0, −0.076), (0.342, 0.643, 0.473) and (0.495, −0.638, 0.320). The red dots indicate the projected positions of the source on the scalp surface. **(B)** Relative errors of the music signals with three references compared to the standard signals. **(C)** Correlation coefficients of three references with the standard signals.

Figure 5 have shown an example of four source simulation with the locations were (0.087, 0.859, 0.097), (0, 0, −0.076), (0.342, 0.643, 0.473) and (0.495, −0.638, 0.320). These sources were marked in Figure 5A. Relative errors were calculated and the results were showed in Figure 5B: these of REST method were lower (0.10 ± 0.10) than the others during all the music pieces; while the AR method was 0.95 ± 0.81; the LM method demonstrated highest variance (1.28 ± 1.04) among the three in such source locations. In such situation, AR method demonstrated a quite low coefficient (0.29) to the standard signal in FP1. And REST method was also almost the same with the standard signals; the coefficient of every electrode was 1. For LM reference, the electrodes, O1 and O2, showed low correlations (0.28 and 0.26) to the standard signal, while in FP1 it was 0.89. Four music pieces from the electrode FP1 on the scalp, including the standard signal, REST, AR and LM, were provided as the Supplementary Materials. Listening to these music, it can be found that in standard and REST music at FP1, the four parts of the melody were not the same as the original music, for channel three had been emphasized. However, in music of AR, this channel was not so prominent.

**Figure 6:**
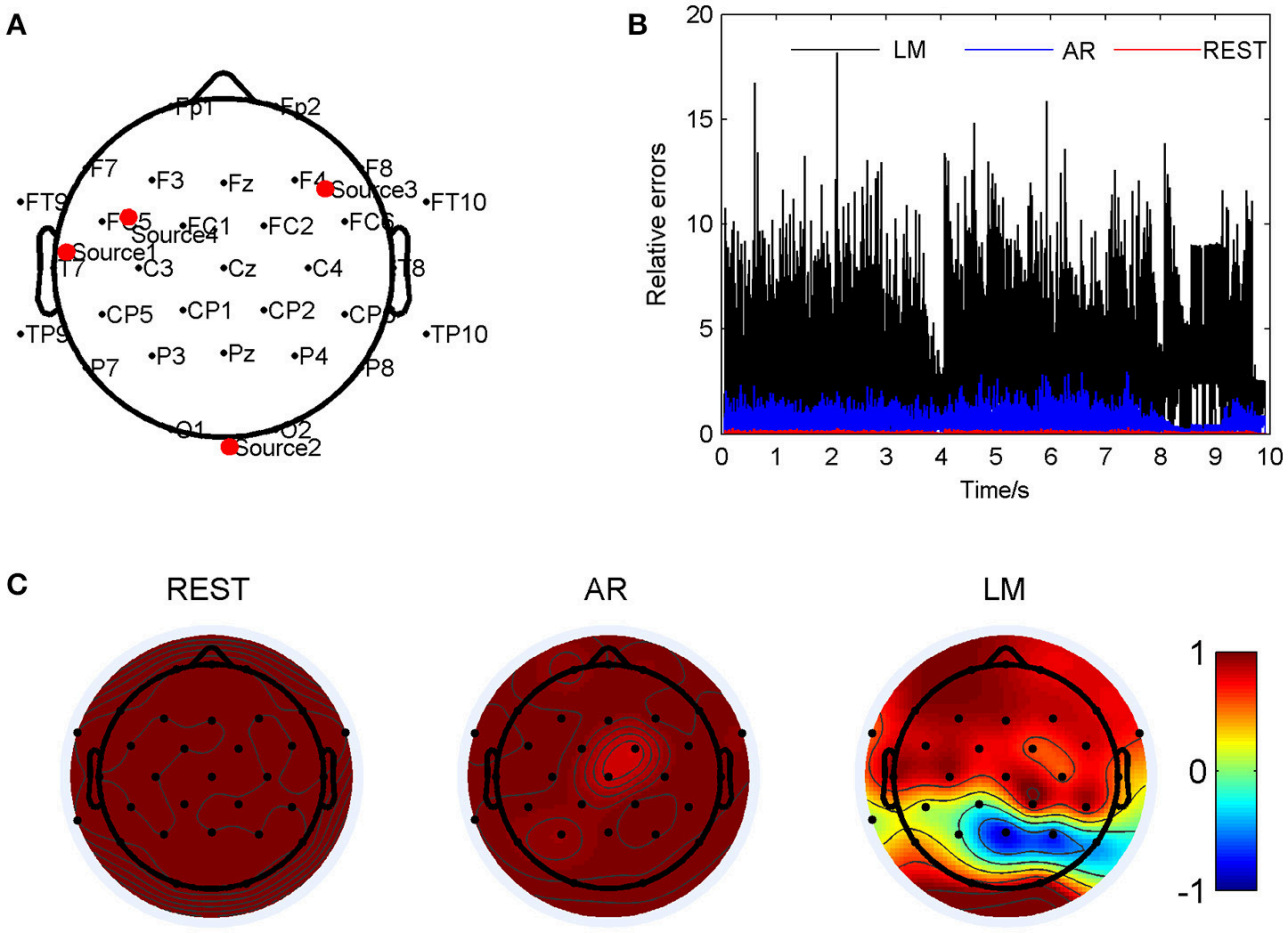
Comparison of the three reference electrode methods for the four sources of K387 shown in Figure 1C. **(A**) The location of the sources are (0.087, 0.859, 0.097), (−0.865, −0.030, −0.076), (0.495, −0.638, 0.320) and (0.342, 0.643, 0.473). The red dots indicate the projected positions of the source on the scalp surface. **(B)** Relative errors of the music signals with three references compared to the standard signals. **(C)** Correlation coefficients of three references with the standard signals.

Another situation of four sources, including (0.087, 0.859, 0.097), (−0.865, −0.030, −0.076), (0.495, −0.638, 0.320) and (0.342, 0.643, 0.473) were represented in Figure 6. The four sources were marked in Figure 6A as red dots. Relative errors were calculated and the results were showed in Figure 6B, we can find that these of REST method were lower (0.05 ± 0.04) than the others during all the music pieces; while the AR method was 0.52 ± 0.41; the LM method demonstrated highest variance (3.34 ± 2.48) among the three in such source locations. In such situation, LM reference demonstrated a quite low coefficient −0.85 and −0.68 to the standard signal in PZ and P4, respectively. And REST method was also almost the same with the standard signals; the coefficient of every electrode was 1. For AR reference, in all the electrodes, showed high correlations (>0.8) to the standard signal, while in PZ it was 0.98. In LM music from the electrode PZ on the scalp, melody of channel four was decreased, and that made the music sounded different from the others.

### Brainwave music from real EEG

The brainwave music generated from real EEG recorded in the pain control experiment was analyzed for the comparison of the references. After EEG segments were translated into quartet music pieces (Wu et al., 2013), there were totally 24 × 3 music sequences from two group's subjects with three references. The music features, note pitch, note duration, tempo, scale free exponent of pitch, and consonance fluctuation were calculated. In MIDI file, the pitch range is from 1 to 127, and 60 represent the middle C. The average pitch of REST was 49.6 ± 10.9; AR was 49.5 ± 11.2; LM was 43.36 ± 10.7. And the differences were significant between REST and LM (*t*-test, *p* < 0.01), AR and LM (*t*-test, *p* < 0.01). However, the REST and AR were not significant different. For all the three references, the differences of music pitch between the two groups were not significant different. The average note duration of REST was 0.71 ± 0.17 s; AR was 0.70 ± 0.15; LM was 0.79 ± 0.18. The REST was significant different from LM (*t*-test, *p* < 0 .01), while AR was significant different from LM (*t*-test, *p* < 0.01). The note duration of REST and AR seem to be similar.

**Figure 7:**
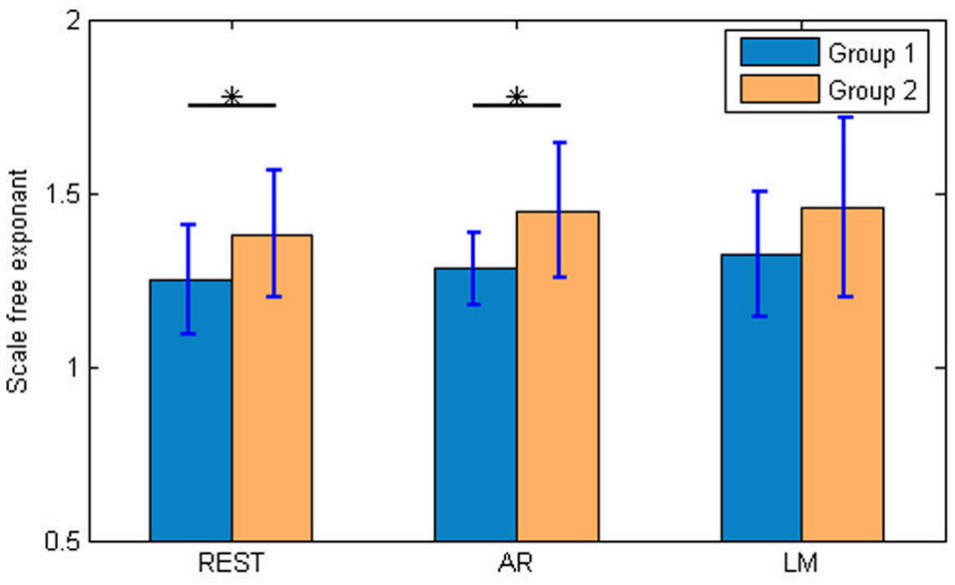
The scale-free exponents of the brainwave music pitch. The scale free exponents of the music pitch were calculated by the Zipf's law. Group 1 was the brainwave music group, while group 2 was the control group. For REST and AR, there were significant differences between the groups. (*t*-test, ^*^p < 0.05).

The scale free exponents of the music pitch are important in our study. The results were showed in Figure 7. The exponents of Group 1 were 1.25 ± 0.16 for REST, 1.28 ± 0.10 for AR, 1.32 ± 0.18 for LM. And for Group 2, REST was 1.38 ± 0.18, AR was 1.45 ± 0.19, and LM was 1.46 ± 0.25. The trend for both two groups was the same: REST < AR < LM. The REST was near 1, which means a more “aesthetic” music, similar with the EEG activities. A two way ANOVA showed significant differences between Group 1 and 2, but no differences between the three references and no interaction of the references and groups. Furthermore, between the two groups, the differences were significant with REST (*t*-test, *p* < 0.05) and AR (*t*-test, *p* < 0.05); while the differences were not significant with LM reference. By listening to the music pieces, it might be easier for distinguishing the two groups when the REST was used.

The consonance fluctuation of these brain music pieces were compared. To calculated the consonance fluctuation, the consonance, which was quantified in terms of the pitch frequency's numerical proportions, at every time point were calculated at first, then the curve of consonance can be obtained, at last, the DFA(detrended fluctuation analysis) were used to analyze the scale exponents of the consonance fluctuation. The more details can be found in my paper of 2015 (Wu et al., 2015). The scale exponents of consonance fluctuation of REST was 0.85 ± 0.05, AR was 0.86 ± 0.04, and LM was 0.88 ± 0.04. The results of REST was significantly different from LM (*t*-test, *p* < 0.05), however the differences between REST and AR, AR, and LM, were not significant.

**Figure 8:**
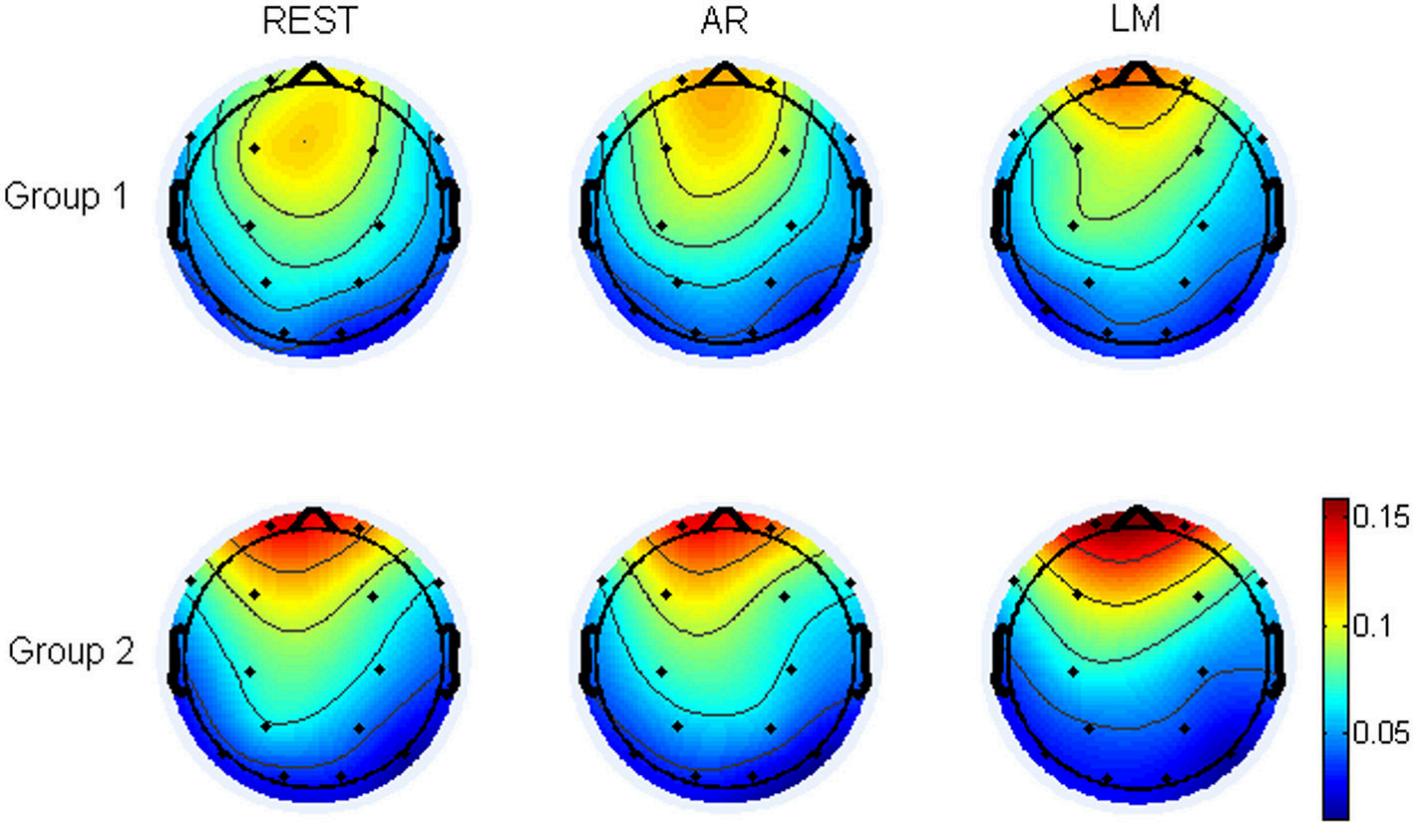
The topographic map of the probability of electrodes being represented for REST, AR and LM references. The topographic map was the average probability for 24 subjects, group 1 (12 subjects) was the brainwave music group, group 2 (12 subjects) was the control group.

Furthermore, the weight of every electrode in the music generation was analyzed. In the translating process of EEG to music, after the data from all the electrodes were changed into music respectively, the notes were selected by a musical filter from the original sequences, so I analyzed the electrodes or regions which were provided more notes in the music generation, which was the weight of electrodes. At last, dividing the number of notes originated from an electrode (weight of electrode) by the total number of notes, I can obtain the probability of electrodes. Figure 8 demonstrated the topographic map of the probability of electrodes being represented for REST, AR, and LM references. The main region of the music generation was the frontal of head in both two groups, though the group 2 (control group) was more concentrated to the frontal. For the distribution of probability, the REST was significant different from AR at electrode P3, and from LM at FP1, FP2, C4, P3, and P4 (*t*-test, *p* < 0.05). These results mean that the frontal region may play an important role in music generation.

The differences of electrodes' probability between the two groups for the three references were also tested respectively. The results showed that for REST, there were significant differences between Group 1 and Group 2 in electrodes FP1, FP2, and T6. And for AR, FP1, FP2, and T6 were also different. Furthermore, FP1, FP2, T6, C4, P3, P4, and O1 were significantly different for LM reference.

## Discussions

We can listen to the brain activities through the brainwave methods proposed previously; whether the music is real reflection of the mental, it depends on whether the data is a true represent of the soul. In the proposed work, I inspect the distortion caused by non-zero reference in the scalp recordings and the corresponding music by simulation and real data. This study draws a picture for us that the “musical” dipoles in the brain generating a piece of music sequence, and then how the different references influence the music we can hear outside the brain. The results of the simulation have shown that the music on the scalp is varied accompanying with the number of the source and distorted by different non-zero reference. When there was more than one source, the scalp music distribution is based on the sources' locations. Therefore, it is important to use a proper reference technology to minimum the errors between real values and the scalp recording values when we plan to preserve the musical information of the EEG.

### From the view of brain signal

Some previous studies compared the three different references method by using simulated dipoles and real EEG data. And the results of simulated data showed that through all the EEG frequency bands including theta, delta, alpha, beta, the features, like power spectra (Trujillo et al., 2017), non-linear features (Chella et al., 2017), the different network connectivity structures, such as default mode network (Qin et al., 2010), the EEG center of mass (Qin et al., 2017), sensor level functional connectivity graphs (Huang et al., 2017), the large-scale brain network (Lei and Liao, 2017), obtained from the REST were more accurate than other references. For real EEG data, there were significant differences existing in the statistics for REST and other references, for example, the ERPs in the audiovisual stimulus, REST changed in the experimental effect (Tian and Yao, 2013). In this study, music is used as the signal source to provide a new view of EEG analysis. Although there are many differences between music waveforms and real EEG data, certain common rhythm exists in the both, i.e., the scale free law (Levitin et al., 2012; Tomasi et al., 2017).

The electrode density and head model are important factors in the reference methods. The previous studies found that the relative error values for Cz and LM references are not noticeably affected by the EEG electrode density. And when electrode density was increasing, the distortion induced by the AR reference may increase (Chella et al., 2017). For the tested montage with 21-channel or 71-channel, REST shows a more reliable reconstruction than AR and LM either with a realistic or a three-layer spherical head model (Liu et al., 2015). Head model is crucial to REST, the more accurate the head model is, the performance of REST is better, so realistic head model usually show better results than the spherical model. However, even in the case of a spherical head model, the REST performance was better than the ones of AR and LM. In this study, 32-channel and the three-concentric-sphere head model were used for simulation, and they are the typical choices in current practices.

The previous studies found that AR reference produced results that were much closer to those of REST, when applied REST, AR and LM references to both simulated and real resting-state EEG data. In the proposed study, the application of the music through the different reference methods demonstrated the same tendency. For most location pairs, REST shows the best, then AR showed high correlation coefficients with the standard signals, and LM is the last one. It means to get the true signal wave, we need to use REST.

### From the view of brain music

It is interesting when some music concepts and analysis are introduced in the study. After the simulation, the music pieces of three references were compared. There were no significant differences between the music on the scalp when there is one singer in the brain (the single source case). Two parts of the music would be restructured on the scalp when there are two singers in the brain, and the variations of the scalp music compared to the inner actual music depend on the position of the two singers and the reference adopted. However, REST is always much better than LM and AR. For four singers in the brain, the phenomena is similar as two singers. Based on simulation, we can recognize the melody because every voice has its own regular. That means the musical conception may be an inspiration of the neuroscience data analysis.

Using music as the source signal have two main advantages. First, it can be recognized very easily through our auditory way when there are some varieties of the original signals. Compared with other generated signals, such as random signal, for simulation, music can be heard when it is analyzed. The distorted music parameters, pitch, volume, tempo, melody, and harmony can be preserved. Second, for two or more sources, it is reasonable that using signals of different music parts as sources. Because each part or voice in the music has its own melody line, but when all of the parts are gathered, they become an integrated work. That is similar to the brain. There may be several active centers or network hubs working separately, and all of them constructing the brain. In the simulation, the comparison of the music parts reveals that the influence of the reference choice when the brain having two or more active sources in a novel perspective.

However, music waveform signals are quite different from real EEG in both frequency and amplitude. To investigate the influence, music pieces may be translated to signals which have the same range of frequency and amplitude of EEG, according to the method proposed in my 2009's paper (Wu et al., 2009). Using these generated EEG as sources for simulation, the comparisons are performed with the three references by calculating the relative errors and coefficients. The details of results are shown in Supplementary Materials. The results indicate that the REST causes the least distortion of the sources, then is AR, and the last is LM. When the EEG sources are put in the same location with the music waveform sources, the coefficients' distributions in the two situations are quite similar (see the figures in Supplementary Materials). However, when using EEG or EEG-like signals as sources, we can't identify the differences between the references by just listening, because the method in Wu's paper (Wu et al., 2009) is mainly for EEG to monophonic music, how to translate EEG into two or four channels is still an open problem.

### For real data exploration

The real EEG data were used as the materials for generating brain music pieces, and the references have caused some differences in some musical parameters. In the exploration, EEG data from two groups were translated into four voices music which is called quartet, according to two main principles: the first is the scale free law that obeyed by both EEG and music (Wu et al., 2009), and the second is a music tone filter method that finding important note and obtaining a sequence with certain tonality (Wu et al., 2013). The references have influenced the musical melodies, for parameters of music represents the features of EEG data. During music generation, EEG amplitude can be indicated by note pitch and EEG period are related to note duration, therefore the data accuracy was one of the most important factors for the data sonified expressing and analyzing. Furthermore, the brain quartet are consisting of notes which picked up by the music tone filter from certain electrodes. The references have also influenced the effects of music filter, making a various note distribution for the quartet. All of these can result in the differences of pitch and duration among the three references.

The scale free exponents of the music are the crucial property of data analyzing, for that is the bridge and connection between EEG and music according to the translation. First, it is interesting that music of REST's exponents are the most close to 1, the next is AR and the last is LM. Previous studies revealed that scale free, of say the 1/f distribution, is a natural feature of human, from the body movement (Torre and Wagenmakers, 2009) to ion channel (Lowen et al., 1999), and brain activity is no exception (Freeman and Breakspear, 2007; Palva et al., 2013). In music, scale free law exists in pitch, tempo, rhythm, and even harmony. The brain music obtained in the experiment maintains such property during the translation from EEG to music note. If it is believed that the brain music indicates the intrinsic rhythm of brain, REST has provided the most faithful signals on the scalp because of its most standard exponents. Second, the exponents of scale free are sometimes regarded as an index for states identification, for example, the sleep stages. In our experiment, the difference between two groups are identified by REST and AR, not the LM. Such results reflect the effects of references on comparing data. Consequently, more accurate data can turn out more useful and meaningful results in data analysis.

The weight of every electrode which were showed in Figure 8 demonstrate the proportion of information provided by each electrode in the music. The filtering criterions, determining which electrodes to be expressed, are derived from the music theory. These criterions make the most stable rhythm and important activities to be represented in the melody. The electrode's weight of three references are different. Although both the brain music group and control group are focused on the frontal region, the distribution of REST can be found with more expressing of the central and occipital region especially in group 1. Such variability makes the identification of the two group more easily.

## Conclusion

The simulation of music as signal sources in the brain indicates that the references deduce the different scalp music which has been heard. The REST showed the smallest relative errors compared to the AR and LM references, and the highest correlation coefficients with the standard sources. The results of real EEG data proved that REST can provide more accurate and natural topographies on the scalp to represent the inner activities. As an inspiration of the neuroscience data analysis, hearing sound in the brain reveals some essential properties with the help of REST.

## Author contributions

DW conceived and designed the study, acquired the data, performed the analysis, interpreted the results, wrote the manuscript and critically reviewed the manuscript.

### Conflict of interest statement

The author declares that the research was conducted in the absence of any commercial or financial relationships that could be construed as a potential conflict of interest.

## Supplementary material

The two original MIDI music and 20 pieces of music (^*^.wav) were provided as Supplementary Material.
