# Supplementary material for: Hearing the Sound in the Brain: Influences of Different EEG References
Source: Front Neurosci. 2018 Mar 13;12:148. doi: 10.3389/fnins.2018.00148 (PMC5859362; doi:10.3389/fnins.2018.00148)
Supplement: Supplementary file 1 [file DataSheet1.DOCX]

Supplementary Material

Hearing the sound in the Brain: Influences of Different EEG References

Dan Wu*

*** Correspondence:** Dan Wu: wudan@bjtu.edu.cn

# Supplementary Audio

Supplementary Audio 1: The standard music at CP1 for one source simulation at (0.087, 0.859, 0.097). The source music was BWV772.

Supplementary Audio 2: The music of REST at CP1 for one source at (0.087, 0.859, 0.097). The source music was BWV772.

Supplementary Audio 3: The music of AR at CP1 for one source at (0.087, 0.859, 0.097). The source music was BWV772.

Supplementary Audio 4: The music of LM at CP1 for one source at (0.087, 0.859, 0.097). The source music was BWV772.

Supplementary Audio 5: The standard music at T7 for two sources at (-0.264, 0.352, 0.750) and (0.278, 0.457, 0.689). The source music was BWV772.

Supplementary Audio 6: The music of REST at T7 for two sources at (-0.264, 0.352, 0.750) and (0.278, 0.457, 0.689). The source music was BWV772.

Supplementary Audio 7: The music of AR at T7 for two sources at (-0.264, 0.352, 0.750) and (0.278, 0.457, 0.689). The source music was BWV772.

Supplementary Audio 8: The music of LM at T7 for two sources at (-0.264, 0.352, 0.750) and (0.278, 0.457, 0.689). The source music was BWV772.

Supplementary Audio 9: The standard music at FP1 for four sources at (0.087, 0.859, 0.097), (0, 0, -0.076), (0.342, 0.643, 0.473) and (0.495, -0.638, 0.320). The source music was K387.

Supplementary Audio 10: The music of REST at FP1 for two sources at (0.087, 0.859, 0.097), (0, 0, -0.076), (0.342, 0.643, 0.473) and (0.495, -0.638, 0.320). The source music was K387.

Supplementary Audio 11: The music of AR at FP1 for two sources at (0.087, 0.859, 0.097), (0, 0, -0.076), (0.342, 0.643, 0.473) and (0.495, -0.638, 0.320). The source music was K387.

Supplementary Audio 12: The music of LM at FP1 for two sources at (0.087, 0.859, 0.097), (0, 0, -0.076), (0.342, 0.643, 0.473) and (0.495, -0.638, 0.320). The source music was K387.

# Supplementary simulation and results

**2.1 Generation for EEG-like sources**

In my 2009’s paper (Wu et al., 2009), a method which can translate EEG signals into music pieces was proposed. The period of EEG was translated to music note duration, and amplitude was converted into music pitch. In this paper, an inverted method which can convert the music melody into EEG signals was also proposed to investigate the influence of the translation. In this study, the inverted method is used to translate the classic music pieces into signals like EEG, which means they have the same frequency and amplitude range. The example of the EEG-like sources are shown in Supplementary Figure 1.

**2.2 Results of one source**

The signal used as a single source in the simulation was *BWV 772* (Figure 1(A)) that composed by Bach. The source was put in 300 different locations of the head modal, and I calculated the relative errors between the standard signals and the re-reference. The average relative errors of the 300 locations were 0.72±2.88 (REST), 4.93±16.69 (AR), and 4.39±7.79 (LM). There were significant differences between REST and AR (t test, p<0.01), REST and LM (t test, p<0.01).

As an example, the coordinate of one source location was (0.087, 0.859, 0.097), and marked by a red dot in Supplementary Figure 2(A). Relative errors of the three different electrode references and correlation coefficients were showed in Supplementary Figure 2(B) and (C), respectively.

**2.3 Results of two sources**

Testing 20*19 pairs of sources, which were chosen from all the 3000 locations according to the head modal, it can be found that the waveform/music showed distinct differences in the three references. The average relative errors of the 380 pairs were 0.17±0.26 (REST), 2.29±1.71 (AR), and 4.12±3.18 (LM). There were significant differences between REST and AR (t test, p<0.01), REST and LM (t test, p<0.01), AR and LM (t test, p<0.01).

Supplementary Figure 3 has shown the situation with source locations at (-0.264, 0.352, 0.750) and (0.278, 0.457, 0.689). The two sources were marked as Source 1 and Source 2 in Supplementary Figure 3(A). Relative errors of the three different electrode references and correlation coefficients were showed in Supplementary Figure 3(B) and (C), respectively.

Supplementary Figure 4 has shown the situation when the two source were at (0.254, 0.124, 0.822) and (-0.678, -0.267, 0.473). The two sources were marked as Source 1 and Source 2 in Supplementary Figure 4(A). Relative errors of the three different electrode references and correlation coefficients were showed in Supplementary Figure 4(B) and (C), respectively.

**2.4 Results of four sources**

360 pairs of electrodes which were chosen from all the 3000 locations of the head modal were tested in the four source simulation, and the average relative errors were 0.17±0.06 (REST), 2.49±0.78 (AR), and 5.20±2.73 (LM), respectively. There were significant differences between REST and AR (t test, p<0.01), REST and LM (t test, p<0.01), AR and LM (t test, p<0.01).

Supplementary Figure 5 have shown an example of four source simulation with the locations were (0.087, 0.859, 0.097), (0, 0, -0.076), (0.342, 0.643, 0.473) and (0.495, -0.638, 0.320). These sources were marked in Supplementary Figure 5(A). Relative errors of the three different electrode references and correlation coefficients were showed in Supplementary Figure 5(B) and (C), respectively.

Another situation of four sources, including (0.087, 0.859, 0.097), (-0.865, -0.030, -0.076), (0.495, -0.638, 0.320) and (0.342, 0.643, 0.473) were represented in Supplementary Figure 6. Relative errors of the three different electrode references and correlation coefficients were showed in Supplementary Figure 6(B) and (C), respectively.

# Supplementary Figures


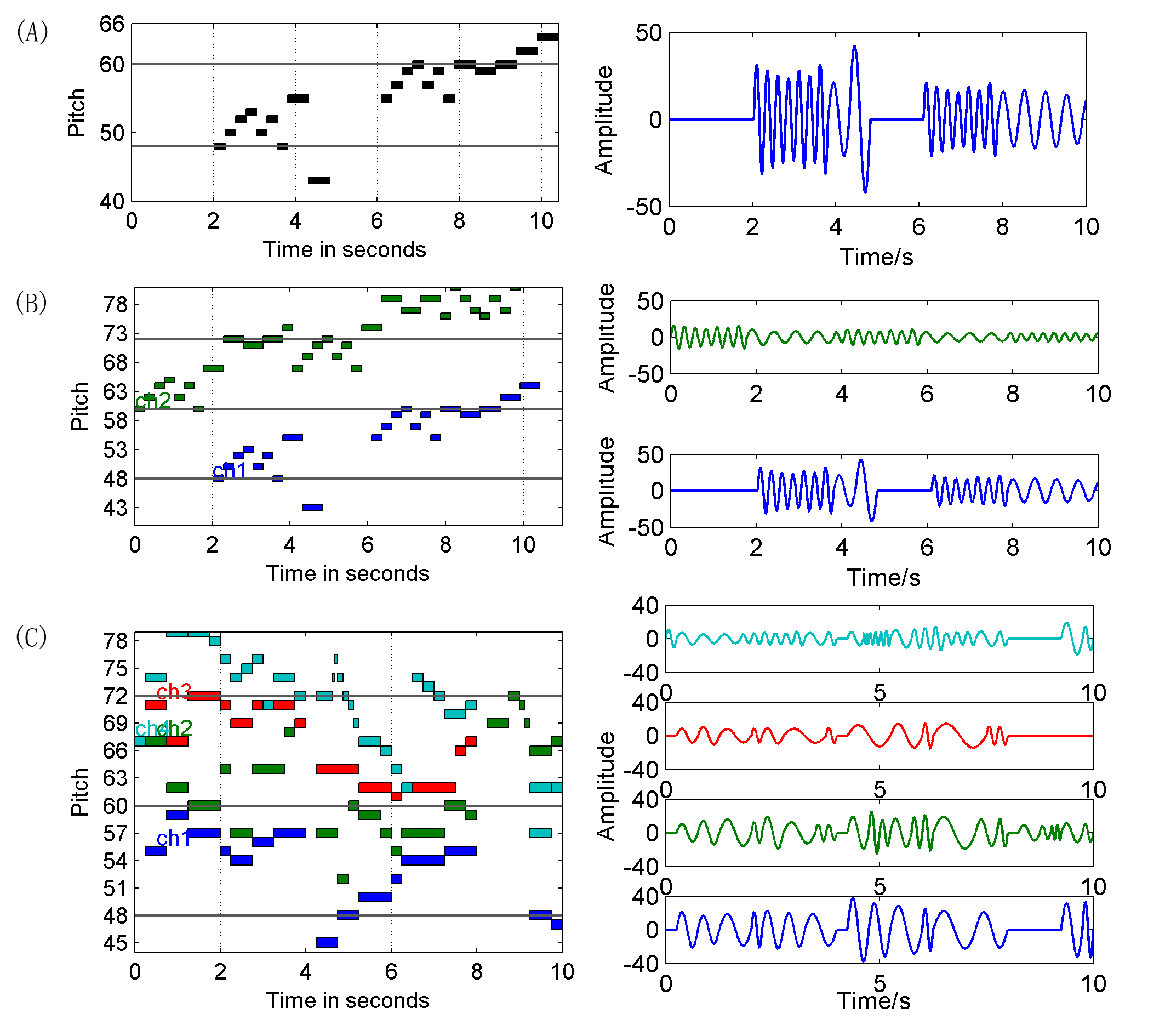


Supplementary Figure 1: Music materials for simulation in MIDI and waveform format (10 seconds for example). (A) MIDI and generated EEG of Bach, BWV 772, with one voice. (B) MIDI and generated EEG of Bach, BWV 772, with two voices. (C) MIDI and generated EEG of Mozart, K387, with four instruments.


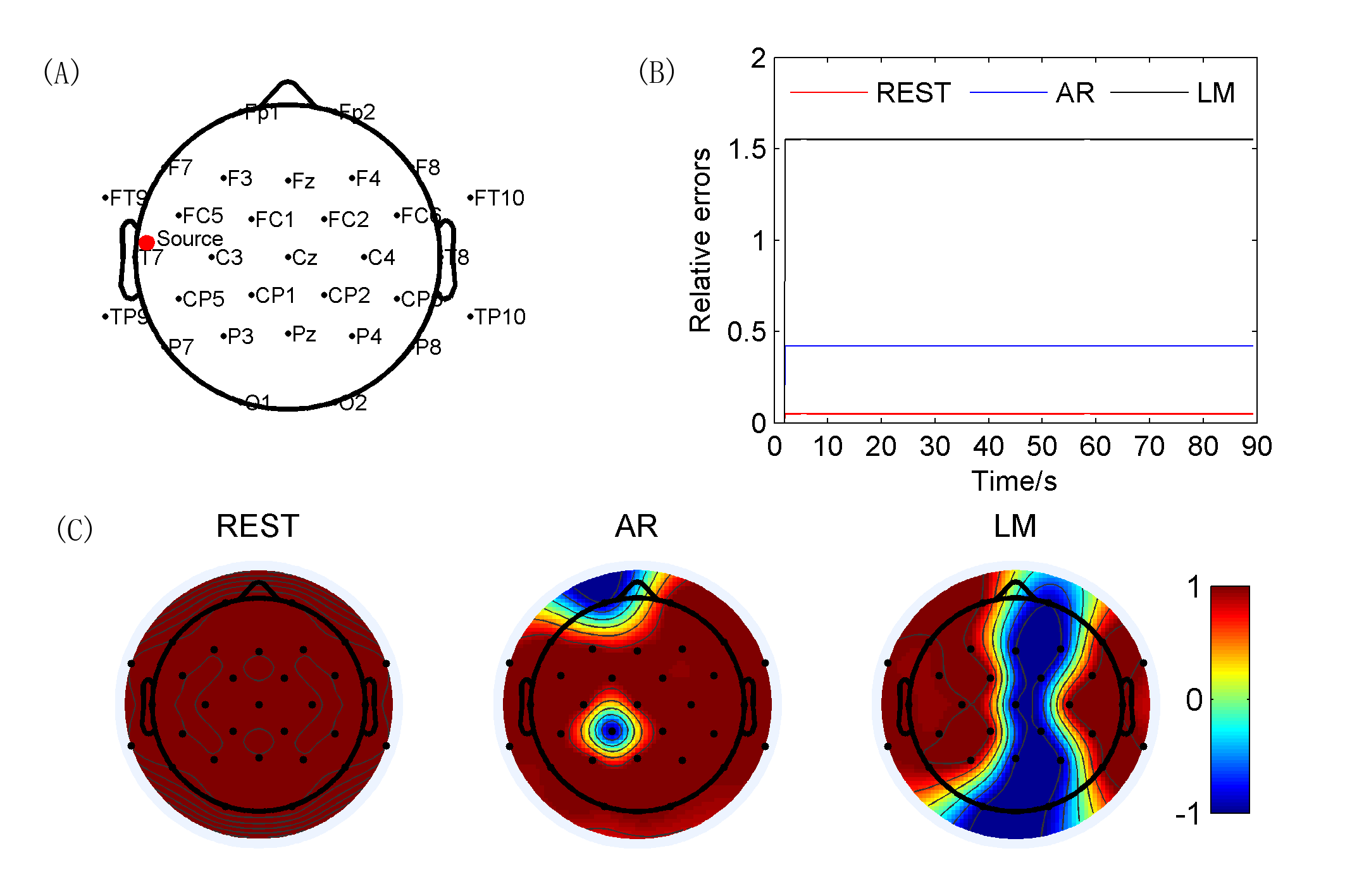


Supplementary Figure 2. Comparison of the three reference electrode methods for the one source of BWV772 shown in Figure 1(A). (A) The location of the source is (-0.65674, -0.06352, 0.56552). The red dot indicates the projected position of the source on the scalp surface. (B) Relative errors of the music signals with three references compared to the standard signals. (C) Correlation coefficients of three references with the standard signals.


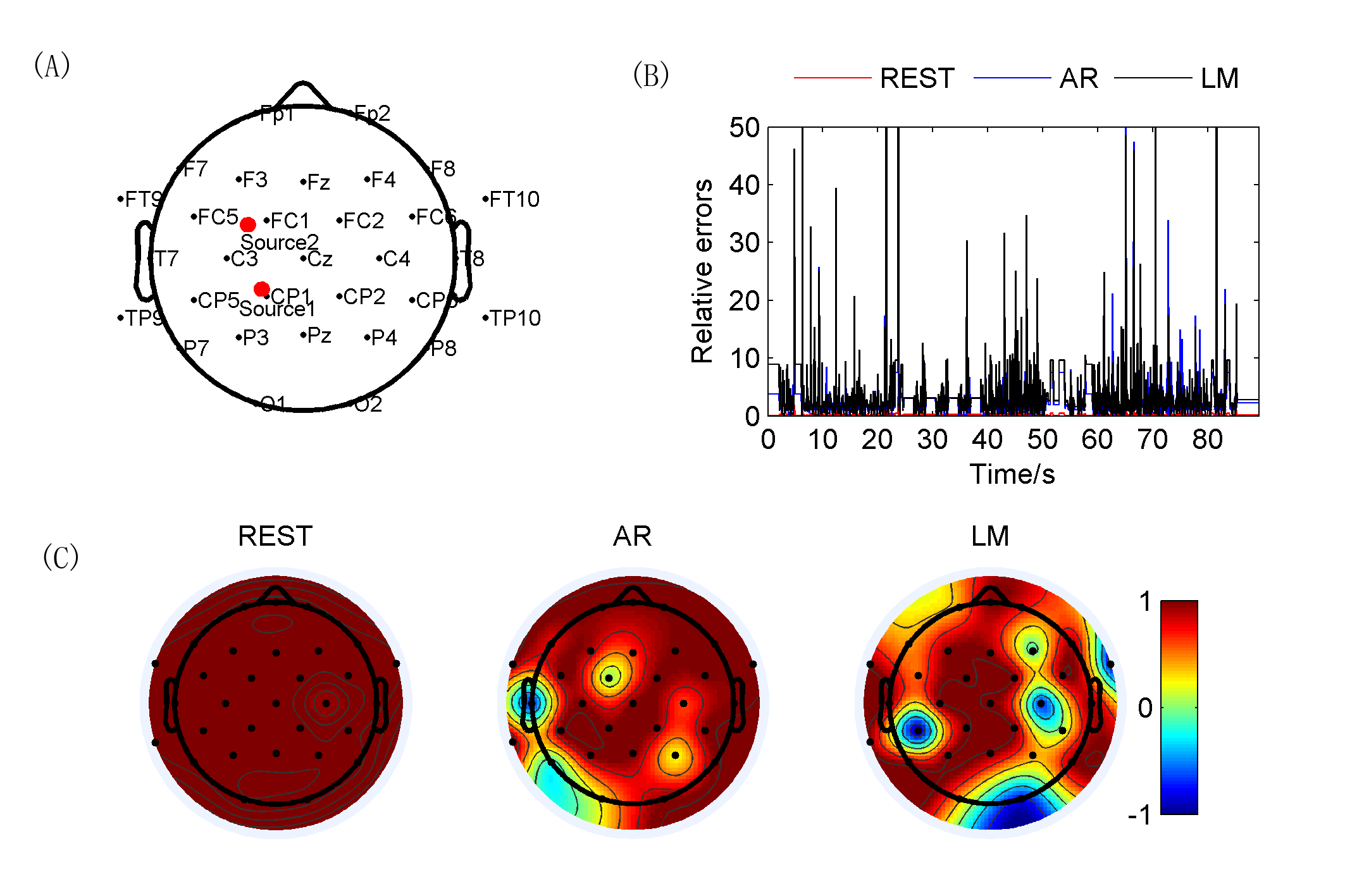


Supplementary Figure 3. Comparison of three reference electrode methods for the two sources of BWV 772 shown in Figure1 (B). (A) The location of the sources are (-0.264, 0.352, 0.750) and (0.278, 0.457, 0.689). The red dots indicate the projected positions of the source on the scalp surface. (B) Relative errors of the music signals with three references compared to the standard signals. (C) Correlation coefficients of three references with the standard signals.


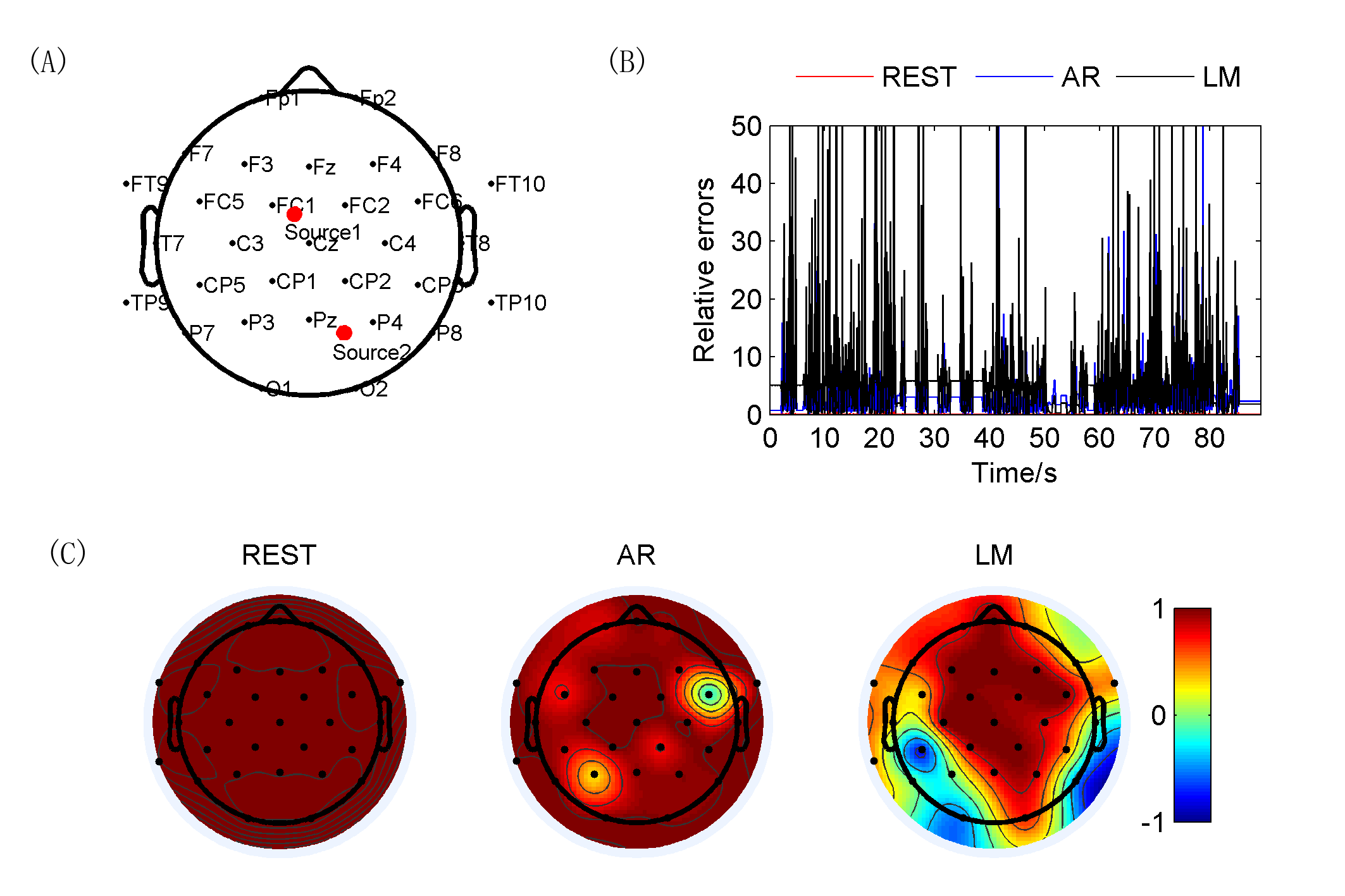


Supplementary Figure 4. Comparison of the three reference electrode methods for the two sources of BWV 772 shown in Figure1 (B). (A) The location of the sources are (0.254, 0.124, 0.822) and (-0.678, -0.267, 0.473). The red dots indicate the projected positions of the source on the scalp surface. (B) Relative errors of the music signals with three references compared to the standard signals. (C) Correlation coefficients of three references with the standard signals.


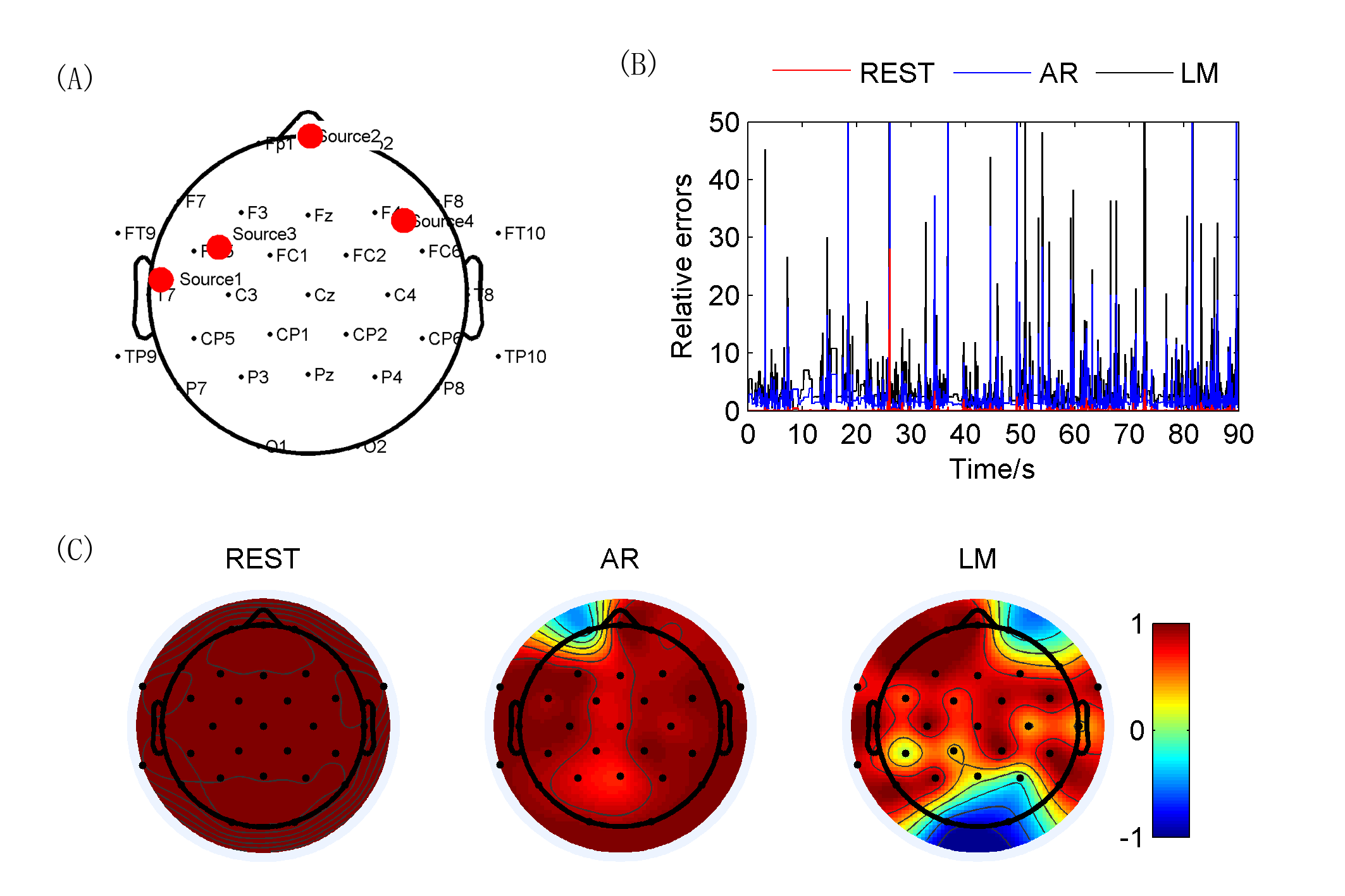


Supplementary Figure 5. Comparison of the three reference electrode methods for the four sources of K387 shown in Figure1 (C). (A) The location of the sources are (0.087, 0.859, 0.097), (0, 0, -0.076), (0.342, 0.643, 0.473) and (0.495, -0.638, 0.320). The red dots indicate the projected positions of the source on the scalp surface. (B) Relative errors of the music signals with three references compared to the standard signals. (C) Correlation coefficients of three references with the standard signals.


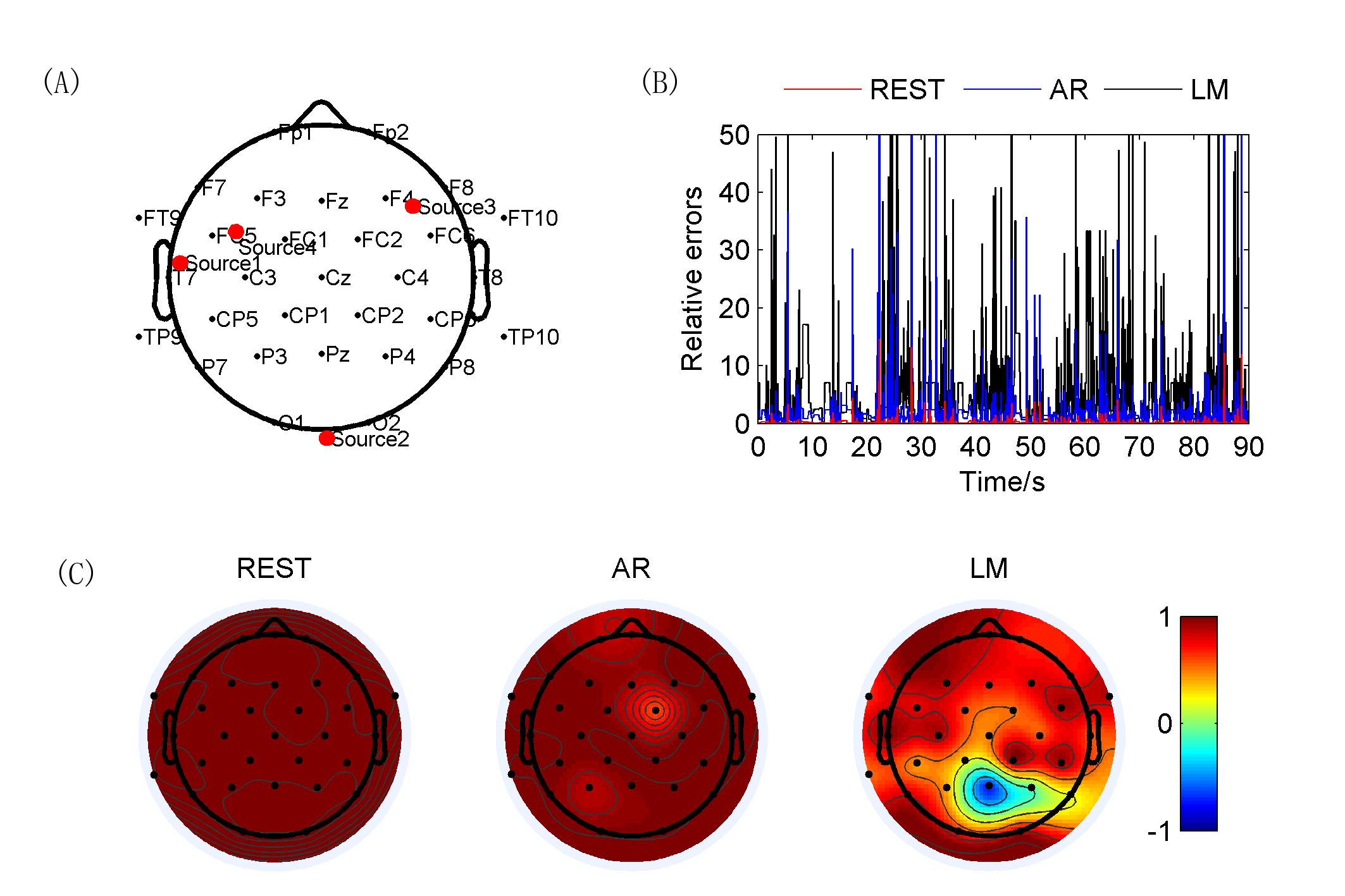


Supplementary Figure 6. Comparison of the three reference electrode methods for the four sources of K387 shown in Figure1 (C). (A) The location of the sources are (0.087, 0.859, 0.097), (-0.865, -0.030, -0.076), (0.495, -0.638, 0.320) and (0.342, 0.643, 0.473). The red dots indicate the projected positions of the source on the scalp surface. (B) Relative errors of the music signals with three references compared to the standard signals. (C) Correlation coefficients of three references with the standard signals.

Wu, D., Li, C., and Yao, D. (2009). Scale-free music of the brain. *PLoS One* 4(6), e5915. doi: 10.1371/journal.pone.0064046.
